# PRO-HOME: AN IMPLEMENTATION SCIENCE FRAMEWORK FOR MULTILEVEL BARRIERS AND FACILITATORS IN LTSS

**DOI:** 10.1093/geroni/igad104.1804

**Published:** 2023-12-21

**Authors:** Katya Cruz Madrid, Michael Berbaum, Surrey Walton, Joseph Zanoni, David Marquez, Naoko Muramatsu

**Affiliations:** University of Illinois Chicago, Chicago, Illinois, United States; University of Illinois Chicago, Chicago, Illinois, United States; University of Illinois Chicago, Chicago, Illinois, United States; University of Illinois Chicago, Chicago, Illinois, United States; University of Illinois Chicago, Chicago, Illinois, United States; University of Illinois Chicago, Chicago, Illinois, United States

## Abstract

Promoting Seniors’ Health with Home Care Aides (Pro-Home) is a randomized controlled trial (RCT) of a gentle physical activity program for frail older adults conducted in a community-based long-term services and supports (LTSS) delivery setting. Pro-Home is a hybrid effectiveness-implementation study, which aims to examine (1) the effectiveness of the program delivered by home care aides (HCAs) in maintaining physical function in older homecare recipients, and (2) how the intervention works in a real-world Medicaid-funded LTSS system. This paper describes barriers and facilitators that have long existed or evolved during the Pro-Home development, using quantitative/qualitative data from the mixed-method study informed by an implementation science framework. Facilitators include the knowledge and partnerships that resulted from multiple preliminary studies that illuminated HCAs’ work/life-related stressors, coping resources, and underutilized capacity and motivation to promote the health of clients and HCAs themselves in the context of the LTSS system. Another facilitator has been the physical activity program developed in another state’s LTSS service setting (not in an academic setting). The program was easy to learn and adapt into a program to be delivered by HCAs (as opposed to care managers). Barriers included turbulent LTSS environments (e.g., rapidly growing managed care presence, COVID-19). Those barriers have been addressed through partnership with stakeholders at multiple levels. Having completed the intervention and data collection phases, we found the project’s planning and implementation challenging but rewarding. Pro-Home has produced rich information that will have many implications for research, practice, and LTSS services delivery and financing systems.

